# Formation and electronic properties of palladium hydrides and palladium-rhodium dihydride alloys under pressure

**DOI:** 10.1038/s41598-017-02617-z

**Published:** 2017-06-14

**Authors:** Xiao Yang, Huijian Li, Rajeev Ahuja, Taewon Kang, Wei Luo

**Affiliations:** 1 Materials Theory Division, Department of Physics and Astronomy, Uppsala University, Uppsala, S75121 Sweden; 2 College of Civil Engineering and Mechanics, Yanshan University, Qin Huangdao, Hebei 066004 China; 3 Department of Materials and Engineering, Royal Institute of Technology (KTH), 10044 Stockholm, Sweden; 4 Nano Information Technology Academy, Dongguk University, Seoul, 100715 Republic of Korea

## Abstract

We present the formation possibility for Pd-hydrides and Pd-Rh hydrides system by density functional theory (DFT) in high pressure upto 50 GPa. Calculation confirmed that PdH_2_ in face-centered cubic (fcc) structure is not stable under compression that will decomposition to fcc-PdH and H_2_. But it can be formed under high pressure while the palladium is involved in the reaction. We also indicate a probably reason why PdH_2_ can not be synthesised in experiment due to PdH is most favourite to be formed in Pd and H_2_ environment from ambient to higher pressure. With Rh doped, the Pd-Rh dihydrides are stabilized in fcc structure for 25% and 75% doping and in tetragonal structure for 50% doping, and can be formed from Pd, Rh and H_2_ at high pressure. The electronic structural study on fcc type Pd_*x*_Rh_1−x_H_2_ indicates the electronic and structural transition from metallic to semi-metallic as Pd increased from x = 0 to 1.

## Introduction

As it is well known that metal hydrides are very interesting systems because of their favourable characteristics including hydrogen-storage capacity, kinetics, toxicity, cyclic behaviour, pressure and thermal response^1^. Especially, the hydrides of platinum group metals are highly attractive due to a number of favourable properties. For instance, the hydrogen absorption by palladium is reversible and therefore has been investigated for hydrogen storage^2^, the catalytic properties, kinetic reversibility^3^ and superconductivity^4, 5^ of palladium hydrides also have been investigated.

It has been reported that hydrogen atoms randomly occupy the octahedral interstices in the Pd-metal lattice with neutron diffraction studies. The limit of absorption at normal pressures is PdH_0.7_, indicating that approximately 70% of the octahedral holes are occupied^6^. Hydrogen absorption in Rh requires extremely high hydrogen pressures (of the order of GPa)^7^ and under normal conditions this metal can only adsorb hydrogens on the surface. Recently, rhodium dihydride was discovered as a first dihydride compound in the platinum group metals by compressing rhodium in fluid hydrogen^8^. The mechanical stability, thermodynamic and elastic properties of RhH_2_ were also studied^9^. With the discovery of RhH_2_, the dihydride of platinum group metals with tetrahedral sites occupied structure was considered to construct the dihydrides of palladium and Pd-Rh-H system alloys.

It is known that the addition of a second metal to palladium changes hydrogen absorption properties of system. It is a consequence of the alteration of crystal lattice structure, elastic and electronic properties^10–13^. Among various Pd alloys, the Pd-Rh system is an exceptional system because the amount of absorbed hydrogen in Pd-rich Pd-Rh alloys is larger than in case of pure Pd^14^. This is in contrast to the general rule that Pd alloys with a non-absorbing metal (e.g., Au, Ag and Pt) are characterised by a decrease in the maximum amount of absorbed hydrogen^15^. An Pd-Rh alloy containing 92.6 at.% Pd has been characterised by the highest hydrogen absorption capacity with H/M ratio exceeding 0.80^16^ using cyclic voltammetry and chronoamperometry in acidic solution.

In this work, we have calculated the formation enthalpy of the hydrides (mono-, di- and tri-) of palladium and rhodium and also Pd-Rh dihydride alloys using DFT approach under high pressure to study the formation possibility. The electronic structure of Pd-Rh dihydride alloys are also analysed by total and partial density of states calculation. The concentration of Pd in Pd-Rh dihydride system alloys is 25%, 50% and 75%, respectively.

## Results and Discussion

### Crystal Structure

**Figure 1:**
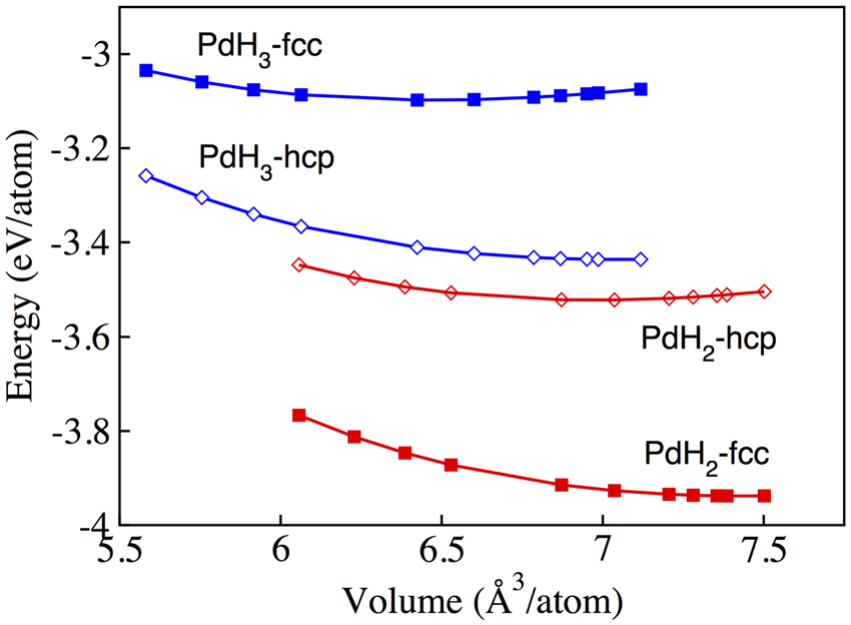
Total energy as a function of volume of fcc and hcp phases for both PdH_2_ and PdH_3_.

The total energy of Pd-Rh-H compounds as a function of volume are shown in Fig. 1. The results show that the PdH_2_ compound in fcc phase is energetically more stable than in hcp phase for the volume range from 7.5 *Å* ^3^/atom to 6.1 *Å* ^3^/atom. PdH_3_ in hcp phase is more stable than fcc phase for the volume range of 7.1 *Å* ^3^/atom to 5.6 *Å* ^3^/atom.

In our calculations, the metal hydride are stabilized in fcc and hcp structures. For monohydride compound PdH, the crystal structure is fcc in space group Fm3m and the hydrogen atom resides in a octahedral sites. For dihydride compounds PdH_2_ and RhH_2_, the crystal structures are same with monohydrides, but the hydrogen atom resides the tetrahedral sites. The trihydride compound RhH_3_ is stabllized in fcc structure, in which the hydrogen atoms are occupied in both tetrahedral and octahedral sites. but PdH_3_ is stabllized in hcp structure in space group of P6_3_/mmc.

Table 1 shows the lattice parameters and bulk modulus of all the compounds compared with experimental data. The bulk modulus are obtained by fitting B-M equation of state with fixed B_0_′ = 4. The bulk modulus B_0_ for Rh-hydrides are larger than Pd-hydrides. As the insertion of H in the octahedral sites of Pd leads to an expansion of the lattice constant from Pd to PdH, the bulk modulus are increased as hydrogen concentration increased. With Rh doped, the bulk modulus of Pd_*x*_Rh_1−*x*_H_2_ are increased with various Rh concentration from 0% to 100%.

**Table 1:** Crystal structure properties of Rh, Pd, RhH, PdH, Pd_*x*_Rh_1−*x*_H_2_, RhH_3_ and PdH_3_ together with available experimental data.

Compounds	Space Group	Lattice constant (*Å*)		B_0_(GPa)	
		Present work	Expt.	Present work	Expt.
Rh	${\rm{Fm}}\tilde{{\rm{3}}}{\rm{m}}$	3.8327	3.8031^21^	264.5	270.4^22^
Pd	${\rm{Fm}}\tilde{{\rm{3}}}{\rm{m}}$	3.9486	3.8898^21^	171.7	180.8^22^
RhH	${\rm{Fm}}\tilde{{\rm{3}}}{\rm{m}}$	4.040(8)	4.020^23^	233.9	
PdH	${\rm{Fm}}\tilde{{\rm{3}}}{\rm{m}}$	4.134(7)	4.090^24^	177.0	130.0^25^ (PdH_0.7_)
RhH_2_	${\rm{Fm}}\tilde{{\rm{3}}}{\rm{m}}$	4.3583	4.3395^8^	190.8	194.3^*h*^
Pd_0.25_Rh_0.75_H_2_	${\rm{Fm}}\tilde{{\rm{3}}}{\rm{m}}$	4.3583	4.3395^8^	175.9	
Pd_0.5_Rh_0.5_H_2_	P4/nbm	a = 4.3167, c/a = 1.05		165.0	
Pd_0.75_Rh _0.25_H_2_	${\rm{Fm}}\tilde{{\rm{3}}}{\rm{m}}$	4.4429		158.0	
PdH_2_	${\rm{Fm}}\tilde{{\rm{3}}}{\rm{m}}$	4.4701		151.5	
PdH_2_	P6/mmm	a = 2,9485, c/a = 0.94		159.2	
RhH_3_	${\rm{Fm}}\tilde{{\rm{3}}}{\rm{m}}$	4.5220		182.9	
PdH_3_	P6_3_/mmc	a = 3.0785, c/a = 2.23		126.4	

### Formation possibility driven by high pressure

**Figure 2:**
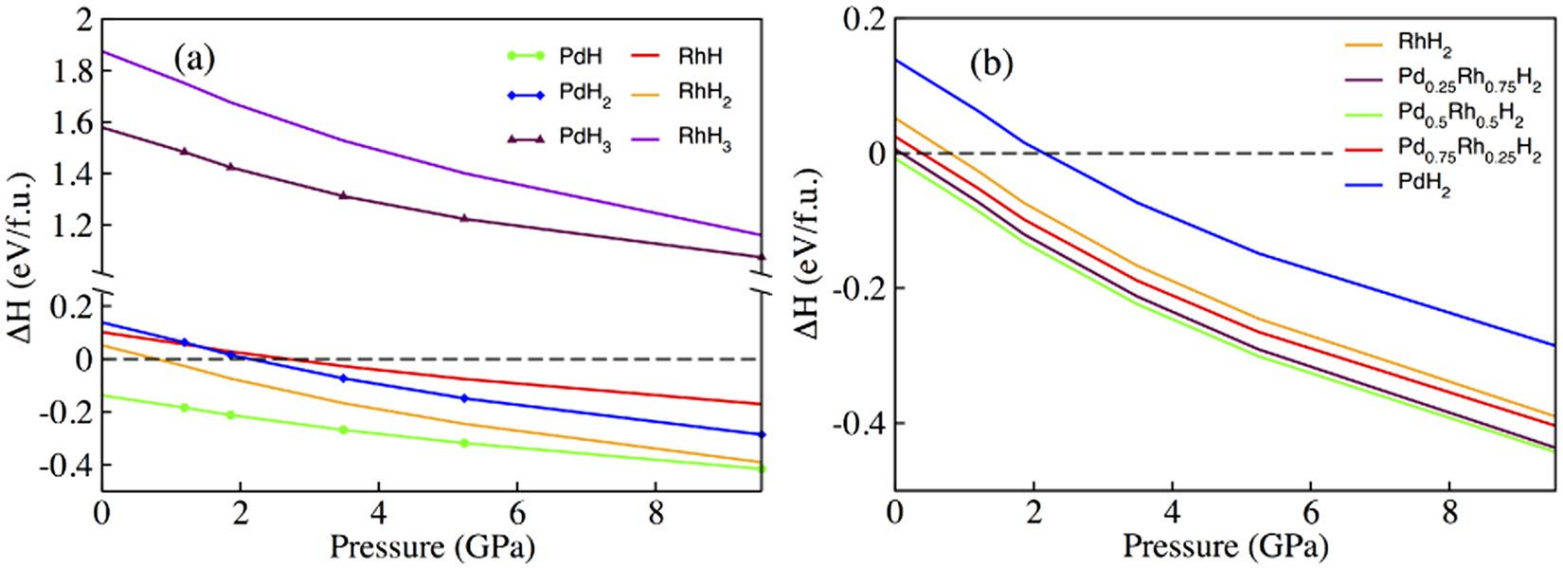
(**a**) The formation enthalpy difference for Pd-H and Rh-H systems. (**b**) The formation enthalpy for Pd_*x*_Rh_1−*x*_H_2_ alloy system. The combined enthalpy of the stable constituent elements establish the reference line corresponding to each compound expressed with dash. ReferenceLine = ∑ [*xH*(*Pd*) + (1 − *x*)*H*(*Rh*) + 2*H*(0.5*H*_2_)].

The enthalpy deferences for Pd-H and Rh-H systems are shown in Fig. 2(a). For PdH and RhH, the enthalpy energy differences is regarding to the chemical reaction equations:

1 $$\begin{array}{l}2PdH=2Pd+{H}_{2}\end{array}$$

2 $$\begin{array}{l}2RhH=2Rh+{H}_{2}\end{array}$$

The PdH has the negative enthalpy of formation, which is consistent with the knowledge that the formation of PdH should be favourable under pressure^17^. The PdH_2_ also should be formed under pressure higher than 2.1 GPa due to the negative enthalpy of formation. The formation properties for RhH and RhH_2_, which both compounds can be formed under pressure, are in good aggreement with the recent work of rhodium dihydride^8^. Whereas for the trihydirde compounds, the positive enthalpy of formation for PdH_3_ and RhH_3_ suggest that they are unfavourable to be formed even with compressing upto 10 GPa.

The formation enthalpy of Pd_*x*_Rh_1−*x*_H_2_ system as a function of pressure is shown in Fig. 2(b). As is shown, the negative enthalpy of formation for Pd_0.5_Rh_0.5_H_2_ in the range of pressure suggests it can be formed even at ambient pressure. While for Pd_0.25_Rh_0.75_H_2_ and Pd_0.75_Rh_0.25_H_2_, the formation enthalpy convert to negative at 0.1 GPa and 0.4 GPa, respectively. Therefore they are more favour to be formed when pressure respectively above 0.1 GPa and 0.4 GPa. Besides, with pressure increasing, the decrease trend of negative formation enthalpy for Pd_*x*_Rh_1−*x*_H_2_ suggests they are more likely to be formed with compressing.

The enthalpy difference of Pd-Rh-H were carried out in total enthalpy between production compound Pd_*x*_Rh_1−*x*_H_2_ and the sum enthalpy of reaction compounds Pd, Rh, and H_2_:

3 $${\rm{\Delta }}E=E(P{d}_{x}R{h}_{1-x}{H}_{2})-[xE(Pd)+\mathrm{(1}-x)E(Rh)+E({H}_{2})]$$

Consider the reaction of Pd and H_2_, PdH as a product of reaction, will compete with PdH_2_ in all range of pressure. To make a further investigation, three reaction paths of PdH_2_ are figured out which respectively is

4 $$\begin{array}{l}Pd{H}_{2}=Pd+{H}_{2}\end{array}$$

5 $$\begin{array}{l}2Pd{H}_{2}=2PdH+{H}_{2}\end{array}$$

6 $$\begin{array}{l}4Pd{H}_{2}=2PdH+2Pd+3{H}_{2}\end{array}$$

**Figure 3:**
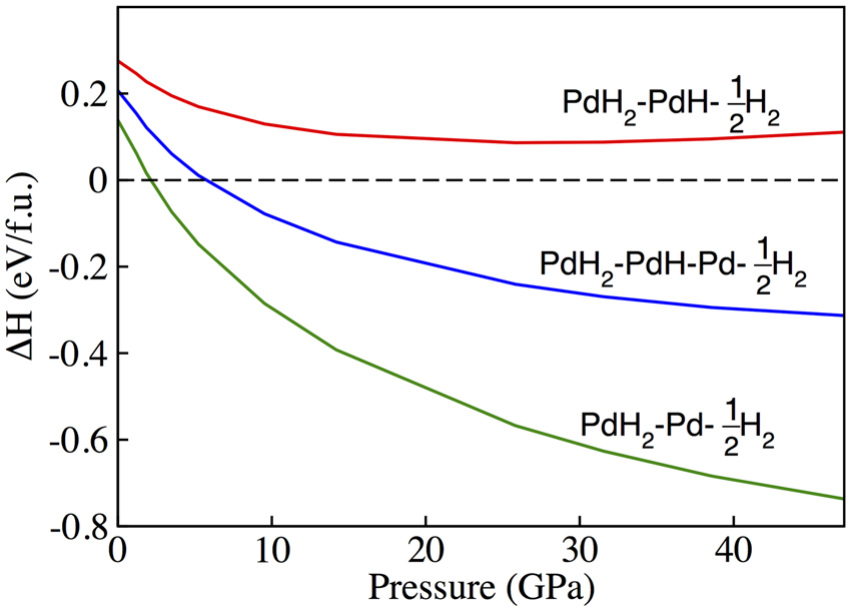
Reaction enthalpy of PdH_2_ according to the three reactions paths: (**a**) PdH_2_ = *Pd* + *H*_2_; (**b**) 2*PdH*_2_ = 2*Pd* + *H*_2_; (**c**) $4Pd{H}_{2}=2PdH+2Pd+3{H}_{2}.$

Figure 3 shows the reaction enthalpy of PdH_2_ with compression upto 50 GPa. The enthalpy of reaction 5 keeps positive in the range of pressure, which suggests PdH and H_2_ is more favourable competing with PdH_2_. Whereas the reaction 6 suggests a decrease trend on the reaction enthalpy with pressure increase, and the reaction enthalpy convert to negative at 5.5 GPa. In this case, the PdH_2_ is more likely to be formed than PdH, Pd and H_2_ when pressure above 5.5 GPa. Therefore, summarising the three reactions above, we conclude that PdH_2_ is metastable and will directly dissociate into PdH and H_2_.

### Electronic Structure Properties

**Figure 4:**
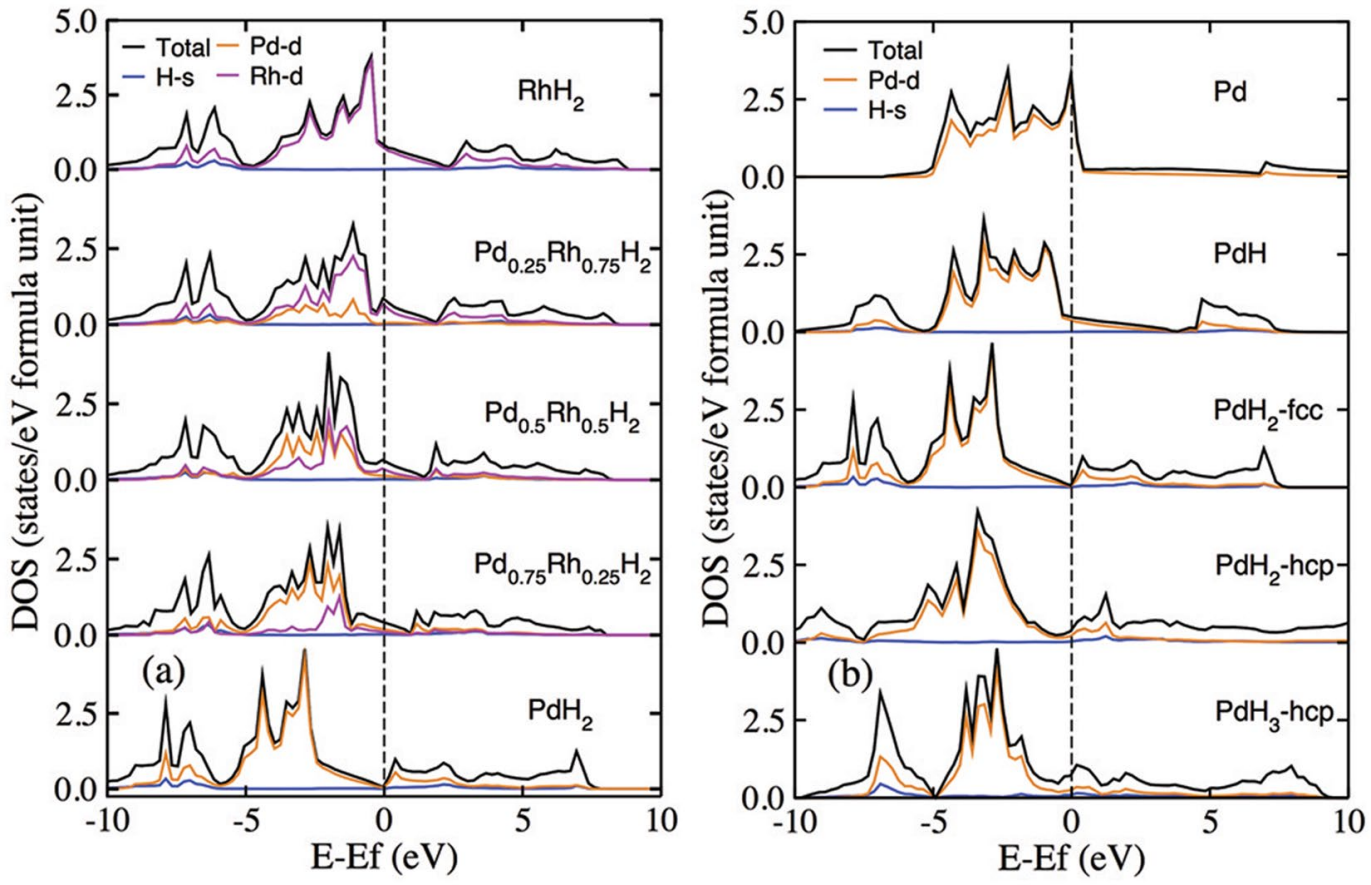
(**a**) Density of state of Pd-Rh-H system at ambient pressure. (**b**) Density of state of palladium hydrides at ambient pressure.

The density of state (DOS) of Pd-Rh-H compounds are calculated at equilibrium volume, as shown in Fig. 4(a). The electronic structure indicated a mixture of metallic and covalent bonding. Below Fermi level there are only occupied states by metal Rh or Pd, and hydrogen electron located in a deeply lower valence band and above fermi level states. In the doped hydrides Pd_*x*_Rh_1−*x*_H_2_, *d*-electron of Palladium shows a strong itinerant electronic properties than Rhodium. By replace 25%, 50%, 75% and 100% Rh atoms with Pd in fcc RhH_2_, the fermi surface shift to lower energy from 8.24 eV for RhH_2_ to 8.06 eV, 7.78 eV, 7.62 eV and 8.03 eV for fcc type PdH_2_, respectively. The DOS of PdH_2_ shows a semimetallic property in which the electronic states around fermi level is less than 0.06.

Figure 4(b) shows the electronic density of states as a function of hydrogen concentration on PdH_*x*_ series while x goes from 0 to 3. In PdH due to the number of H-filled Pd octahedra, the hybridized band H_*s*_/Pd_*d*_ appeared and partially filled on the range of −6.2 ∼ −7.9 eV. In additional, the valence/conduction band on the fermi level is dominated by the 4*d* orbitals of the palladium atoms in PdH, but the fermi level shifted from 8.94 eV for pure Pd to 8.86 eV for PdH. The electronic states on fermi level are decreased from 3.32 to 0.51 states/eV/f.u.

When hydrogen number increases to 2, two structures fcc and hcp PdH_2_ are considered. The dispersion of the density of states for fcc PdH_2_ mainly followed the curve on fcc PdH, in which fermi level shift from 8.86 eV for PdH to 8.03 eV for PdH_2_. The observed difference is the electronic property that changed from metallic to “near insulator” owing to the density of states on fermi level tends to zero. For hcp PdH_2_ that electron dispersion is following the curve on hcp PdH_3_, in which hydrogen s orbital contributes to the valence/conduction band on the fermi level.

## Conclusion

In conclusion, three different types of palladium and rhodium hydrides and Pd-Rh-H dihydride alloys have been investigated by first principle calculation. We have found that PdH_2_ is not stable and dissociate to PdH and H_2_ at ambient and even higher pressure. While when palladium is involved in the reaction, PdH_2_ can be easy formed from lower pressure around 10 GPa. With Rh doping alloy hydrides Pd_*x*_Rh_1−*x*_H_2_ is formed from fcc metal Pd and Rh in H_2_ atmosphere at even lower pressure. The electronic density of states investigations show that the Pd_*x*_Rh_1−*x*_H_2_ has metallic properties whereas PdH_2_ semimetallic property.

## Methods

The DFT calculations are carried out by employing the Vienna Ab-initio Simulation Package(VASP)^18^ implementing the Projector Augmented Wave method. The generalized gradient approximation^19^ was used for the correlation energy function^20^ with the Perdew Burke Ernzerhof parameterisation. The valence electron configurations for Pd and Rh were 4p^6^5 s^1^4d^9^ and 4p^6^5 s^1^4d^8^, respectively. The relaxation convergence for ions and electrons are 10^−2^ and 10^−6^ eV, respectively. The electronic wave function was expanded in a plane wave with an energy cut-off 800 eV. For energy formation calculation, the 24 × 24 × 24 Monkorst-Pack(MP) K mesh for Brillouin zone integration was used for the structure optimisation and static calculation. For DOS calculations, the K mesh was increased to 32 × 32 × 32 for fcc compounds, and 15 × 15 × 7 mesh for hcp PdH_3_, respectively.
